# Muscle Psn gene combined with exercise contribute to healthy aging of skeletal muscle and lifespan by adaptively regulating Sirt1/PGC-1α and arm pathway

**DOI:** 10.1371/journal.pone.0300787

**Published:** 2024-05-16

**Authors:** Ying-hui Gao, Deng-tai Wen, Zhong-rui Du, Jing-feng Wang, Shi-jie Wang

**Affiliations:** Ludong University, City Yantai, Shandong Province, China; The University of Alabama at Birmingham Department of Pathology, UNITED STATES

## Abstract

The Presenilin (Psn) gene is closely related to aging, but it is still unclear the role of Psn genes in skeletal muscle. Here, the Psn-UAS/Mhc-GAL4 system in Drosophila was used to regulate muscle Psn overexpression(MPO) and muscle Psn knockdown(MPK). *Drosophila* were subjected to endurance exercise from 4 weeks to 5 weeks old. The results showed that MPO and exercise significantly increased climbing speed, climbing endurance, lifespan, muscle SOD activity, Psn expression, Sirt1 expression, PGC-1α expression, and armadillo (arm) expression in aged Drosophila, and they significantly decreased muscle malondialdehyde levels. Interestingly, when the Psn gene is knockdown by 0.78 times, the PGC-1α expression and arm expression were also down-regulated, but the exercise capacity and lifespan were increased. Furthermore, exercise combined with MPO further improved the exercise capacity and lifespan. MPK combined with exercise further improves the exercise capacity and lifespan. Thus, current results confirmed that the muscle Psn gene was a vital gene that contributed to the healthy aging of skeletal muscle since whether it was overexpressed or knocked down, the aging progress of skeletal muscle structure and function was slowed down by regulating the activity homeostasis of Sirt1/PGC-1α pathway and Psn/arm pathway. Exercise enhanced the function of the Psn gene to delay skeletal muscle aging by up regulating the activity of the Sirt1/PGC-1α pathway and Psn/arm pathway.

## Introduction

The number of aging populations around the world shows a continuous and rapid growth trend, and most of these older people suffer from the negative effects of aging-induced physical deterioration on their organisms [1]. One of the negative effects of physical deterioration is that it can have a serious impact on the skeletal muscles, causing them to experience a loss of function [2]. Loss of function in skeletal muscle can manifest itself in the form of reduced skeletal muscle weight and size, which are collectively known as sarcopenia [3]. Loss of skeletal muscle function can severely inconvenience the independent living of older persons, leading to a serious decline in their standard of living and placing a serious burden on the world economy in all respects [4]. Therefore, improving skeletal muscle is an important area of anti-aging research.

Presenilin (Psn) is a multichannel transmembrane protein associated with organismal aging that affects multiple molecular mechanisms [5]. Psn is widely expressed during development and is commonly expressed in the tissue distribution of adults [6]. The *Drosophila* model has become a classical model organism widely used in the fields of genetics and development because of its short life cycle, ease of cultivation, relative maturity of transgenic technology, and the fact that it contains at least 75% of the homologous genes associated with human diseases. [7]. *Drosophila* has two Psn homologs, presenilin-1 (PS1) and presenilin-2 (PS2), which are both genes for proteins with anti-apoptotic effects, with PS1 being the more important one [8]. PS1 can regulate the cell cycle through the Wnt/arm pathway and plays an important role in delaying cellular senescence and reducing oxidative stress [9]. PS2 overexpression is associated with apoptosis [10]. Synaptic dysfunction observed in a PS1 overexpressing Alzheimer’s disease cell model [11]. PS1 knockdown results in cell growth injury, decreased viability, and endoplasmic reticulum stress response [12]. Its knockdown in the midgut produces viable *Drosophila* [13]. However, the Psn gene has been less studied in muscle aging, and its role in age-induced decline in skeletal muscle is currently unclear.

Endurance sports workouts can be effective in exerting antioxidant and anti-aging benefits [14]. In both mammals and *Drosophila*, increasing evidence has shown that the use of regular exercise training is an effective prevention and treatment modality in the prevention and treatment of many age-dependent neurodegenerative chronic diseases induced by the aging of the organism [15]. Endurance exercise training can enhance muscle function by increasing the antioxidant capacity of the muscle to delay muscle fatigue as a manifestation of a physiological muscle adaptation [16]. In the study of senile sarcopenia, it has been found that loss of muscle function can be mitigated by increasing physical activity to promote protein synthesis and increase anabolism [17]. Exercise training also enhances muscle mass, reduces the risk of chronic diseases of the metabolic system, and plays an important role in the treatment and prevention of age-dependent neurodegenerative diseases [18]. Notably, a recent study reported that regular exercise could alleviate pathological symptoms and behavioral characteristics. To explore Alzheimer’s disease caused by Psn gene mutations [19]. However, the role of the psn gene and endurance exercise in anti-aging in skeletal muscles is still unclear.

To explore the role of skeletal muscle Psn gene in age-induced skeletal muscle aging and its molecular mechanism, and whether psn regulates the process of exercise to delay skeletal muscle aging. We utilized the Psn-UAS/Mhc-GAL4 system in *Drosophila* to regulate muscle Psn overexpression (MPO) and muscle Psn knockdown (MPK). The *Drosophila* underwent endurance exercises using the *Drosophila* movement device. The muscle Psn, Sirt1, PGC-1α, and armadillo (arm) gene expression levels were tested by quantificational qRT-PCR. And detection of SOD activity levels and malondialdehyde (MDA) levels in Drosophila skeletal muscle by enzyme-linked immunosorbent assay (ELISA) [20]. Based on these metrics, we sought to understand the effects of MPO combined with endurance exercise and MPK combined with endurance exercise on skeletal muscle aging and lifespan and to elucidate the molecular mechanisms.

## Materials and methods

### *Drosophila* strains and feeding

The Psn-UAS-knockdown (Psn-UAS-RNAi) *Drosophila* (stock ID:v43082; FlyBase Genotype: w^1118^; P{GD4624}v43082) obtained from the Vienna *Drosophila* Resource Center. The Psn-UAS-overexpression (Psn-UAS-OE) *Drosophila* (stock ID:8313; FlyBase Genotype:y^1^ w^1118^; P{UAS-Psn.541.M255V}3) and the Mhc-GAL4 *Drosophila* (stock ID:55133; FlyBase Genotype:w*;P{Mhc-GAL4.K}2/TM3, Sb1) were obtained from the Bloomington *Drosophila* Stock Center.“y^1^ w^1118^; P{UAS-Psn.541.M255V}3>Mhc-GAL4” was represented as “Psn-overexpression (Psn-OE)”. “w^1118^; P{GD4624}v43082>Mhc-GAL4” was represented as “Psn-knockdown (Psn-RNAi)”. Age-matched Psn-UAS-OE male *Drosophila* were divided into 2 groups: Psn-UAS-OE group(Psn-UAS-OE) and Psn-UAS-OE+exercise group(Psn-UAS-OE+E); age-matched Psn-OE male *Drosophila* were divided into 2 groups: Psn-OE group (Psn-OE) and Psn-OE+exercise group (Psn-OE+E); age-matched Psn-UAS-RNAi male *Drosophila* were divided into 2 groups: Psn-UAS-RNAi group(Psn-UAS-RNAi) and Psn-UAS-RNAi+exercise group(Psn-UAS-RNAi+E); age-matched Psn-RNAi male *Drosophila* were divided into 2 groups: Psn-RNAi group (Psn-RNAi) and Psn-RNAi+exercise group (Psn-RNAi+E).

During the experimental time course, *Drosophila* medium is prepared with 2% agar, 1% soybean powder, 1.3% yeast, 4.2% corn meal, 0.8% agar, 3.1% sucrose, 3.1% maltose, and 0.2% propionic acid. All *Drosophila* were incubated in an incubator with a suitable environment for the growth of *Drosophila* [21].

All the *Drosophila* were kept without any intervention until the third weekend and then given endurance exercises in the fourth week of their lives.

### Endurance exercise device and protocols

Using the *Drosophila* Locomotion Training Facility, *Drosophila* were made to climb upwards in a test tube for endurance locomotion training by utilizing the anti-gravity climbing characteristic that *Drosophila* possesses [22]. Thirty *Drosophila* per tube were placed horizontally on the *Drosophila* locomotor training facility when the *Drosophila* climbed to the top of the test tube, the test tube was rotated 180° at a uniform speed of 60 rad/s, 8 cm as the *Drosophila* locomotor area and the *Drosophila* were allowed to have a 10s period of time to climb upward. All the locomotor *Drosophila* started locomotor training from the 22nd day of age, 1 hour of locomotion per day, 2 consecutive days of locomotor training per week, and 1 day of rest for 2 weeks. Measurements and samples were taken 24 hours after the end of locomotor training (Table 1).

**Table 1:** Exercise flow chart.

	Monday	Tuesday	Wednesday	Thursday	Friday	Saturday	Sunday
1st week of age	ND	ND	ND	ND	ND	ND	Measure climbing and endurance
2nd week of age	ND	ND	ND	ND	ND	ND	ND
3rd week of age	ND	ND	ND	ND	ND	ND	Measure climbing and endurance
4th week of age	**E**	**E**	ND	**E**	**E**	ND	Measure climbing and endurance
5th week of age	**E**	**E**	ND	**E**	**E**	ND	Measure climbing and endurance

Exercise training program.

Note: ND for no exercise intervention, E for exercise intervention.

### Climbing ability assay

3-Second Climbing Height: At the end of 1, 3, 4, and 5 weeks of age *Drosophila*, respectively, the 3-second climbing height of *Drosophila* was measured by using a SONY video camera to record the height of the *Drosophila* after falling to the surface of the medium by shocking the *Drosophila* due to the feature of climbing against gravity for 3 seconds, and the data were used to reflect the locomotor ability of the *Drosophila*. For each *Drosophila* strain 100 *Drosophila* were randomly selected, and 25 *Drosophila* were placed in each tube, totaling 4 tubes. Before measurement and shooting, the tubes were shaken 3 times to make *Drosophila* fall to the surface of the medium and produce adaptation. When shooting, to ensure the accuracy of the experimental data collection, the fruit *Drosophila* should all fall to the surface of the medium when shaking. Leave 8cm as the range of *Drosophila* movement area, and the height climbed by *Drosophila* in 3 seconds was measured to assess the locomotor ability of *Drosophila* [23].

Climbing endurance: using a facility for *Drosophila* locomotor training, at the end of 1, 3, 4, and 5 weeks of age, 15 *Drosophila* were randomly sampled from each group and divided into 15 tubes with 1 *Drosophila* in each tube. When the *Drosophila* at the bottom of the tube climbed to the top, the tube rotated at a constant speed of 60rad/s, and the *Drosophila* climbed back to the top until the top and bottom swapped, and so on. Reserve 8cm of culture medium from the bottom of the test tube to the bottom of the cotton plug for the *Drosophila* movement area. After the two ends of the test tube change directions, there are 10 seconds for climbing. When *Drosophila* climbed a certain distance after the tube was flipped over and then stopped eventually failing to reach the top or ceasing to climb, the time of *Drosophila* exhaustion was recorded, which we considered to be the maximum climbing endurance of *Drosophila*, and this time was used to evaluate *Drosophila* exercise ability [24].

### ELISA assay

The ELISA assay-specific steps are as follows: 1) Sample addition of standard products. 2) Sample addition: Prepare blank control wells and sample wells to be tested. Add 40 μl of diluent to the sample wells to be tested, and then add 10 μl of test sample. 3) Add enzyme: Add enzyme-labeled reagent 100μl per hole, except blank hole. 4) Incubation: After the sealing plate is sealed with sealing plate film, it is incubated at 37°C for 60 minutes. 5) Mixing liquid: Dilute 20 times concentrated washing liquid with 20 times distilled water for reserve use. 6) Wash: Remove the sealing film from the surface of the sealing plate, pour out the solution in it, clean it, and then let it dry. 7) Color development: Add color-developing agent A50μl to each well first, then add color-developing agent B50μl, gently shake and mix, and hide from light for 15 minutes at 37°C. 8) Termination: Add termination solution 50μl per well to terminate the reaction (at this time, the blue immediately turns to yellow). 9) Determination: Determine the absorbance (OD) of each well.

### qRT-PCR

About 50 *Drosophila*’ muscles from each group were homogenized in Trizol. (1) Total RNA extraction: Take the homogenizer tube, add 1ml of RNA extraction solution, place it on ice for pre-cooling, take 100mg of tissue, add it into the homogenizer tube, and grind it fully with the grinder until no tissue mass is visible. Centrifuge at 12000rpm at 4°C for 10min, white precipitate at the bottom of the tube is RNA. Remove the liquid, add 1.5ml of 75% ethanol for washing and precipitation, centrifuge at 12000rpm at 4°C for 5min, remove the liquid, put the centrifuge tube on a super-clean table for 3min, add 15μl Water Nuclease to dissolve RNA Free, and incubate at 55°C for 5min. Nanodrop 2000 was used to detect RNA concentration and purity: After blank zero adjustment of the instrument, 2.5μl RNA solution to be tested was put on the detection base, the sample arm was lowered, and the software on the computer was used to start the absorption value detection, and the RNA with excessive concentration was diluted in an appropriate proportion so that the final concentration was 100–500 ng/μl. (2) Reverse transcription: Configure the reverse transcription reaction system, gently mix and centrifuge, and set the reverse transcription program. (3) Quantitative PCR: 0.2ml PCR tubes were used to prepare the reaction system. Each retro product was prepared with 3 tubes for PCR amplification. (4) Results treatment: CT method. Primer sequences of Psn were as follows: S: 5′-AATACCTGCCTGAATGGACTGC-3′; A: 5′- AGATTTGCTCATTTCGCTCCTG-3′. Primer sequences of Sirt1 were as follows: S: 5′-ACAGAAGATCATTGTGCTAACGGG-3′; A: 5′-CAAACTTGTAGAACGGTCGTGGAT-3′. Primer sequences of PGC-1α were as follows: S: 5′-ACCTGGCGATTCTGATTATGACT-3′; A: 5′-CCTTTACATTGTCCACATAGCGT-3′. Primer sequences of arm were as follows: S: 5′-GAGGACAAGCCGCAGGATTA-3′; A: 5′-CGTACAGGCCCTCATATGCT-3′. Primer sequences of Rp49 were as follows: F: 5′-CTAAGCTGTCGCACAAATGG-3′; R: 5′-AACTTCTTGAATCCGGTG GG-3′. qRT PCR—measurement of all genes are after a large number of transcription regulation, this related to function.

### Lifespan assays

For each strain of *Drosophila*, 100 *Drosophila* were randomly selected from the 36th day of age. Several deaths were counted every two days at the time of changing the *Drosophila* medium until all the *Drosophila* of each strain were dead, and the survival curve of *Drosophila* was made. The arithmetic mean of the life span of all *Drosophila* was the average life span of *Drosophila*, and the average rate of extended life (%) = (average life span of experiment group—average life span of control group)/average life span of the control group ×100%. The average life span, maximum life span, and average life extension of *Drosophila* were analyzed [25].

### Statistical analyses

To investigate the effects of endurance exercise, differential Psn gene expression, and combined intervention on aging Drosophila, experiments were grouped in a completely randomized design with overexpression/knockdown groups and overexpression/knockdown exercise groups, normal expression groups, and normal expression exercise groups. Data from nine groups were repeatedly measured and compared for differences. The 1-way analysis of variance (ANOVA) with least significant difference (LSD) tests was used to identify differences among these groups. p values for lifespan curves and climbing endurance curves were calculated by the log-rank test. Analyses were performed using the Statistical Package for the Social Sciences (SPSS) version 16.0 for Windows (SPSS Inc., Chicago, USA), with a statistical significance set at p<0.05. Data are represented as means ± SEM.

### Ethics approval

All experimental designs and protocols involving animals were approved by the Animal Ethics Committee of the Ludong University, Yantai, People’s Republic of China (approval LDU-IRB2022030801) and complied with the recommendations of the academy’s animal research guidelines.

## Results

### MPO improves age-related decline in skeletal muscle and lifespan in *Drosophila*

Increasing evidence confirms that the climbing ability of *Drosophila* can be measured by assaying climbing speed and climbing endurance [26]. The Climbing Height (CH) test and Time to Fatigue (TTF) test in Drosophila are both important indicators of skeletal muscle strength and function [27]. In this study, the muscle Psn gene was overexpressed by Psn-UAS-overexpression/Mhc-GAL4 system.

**Fig 1:**
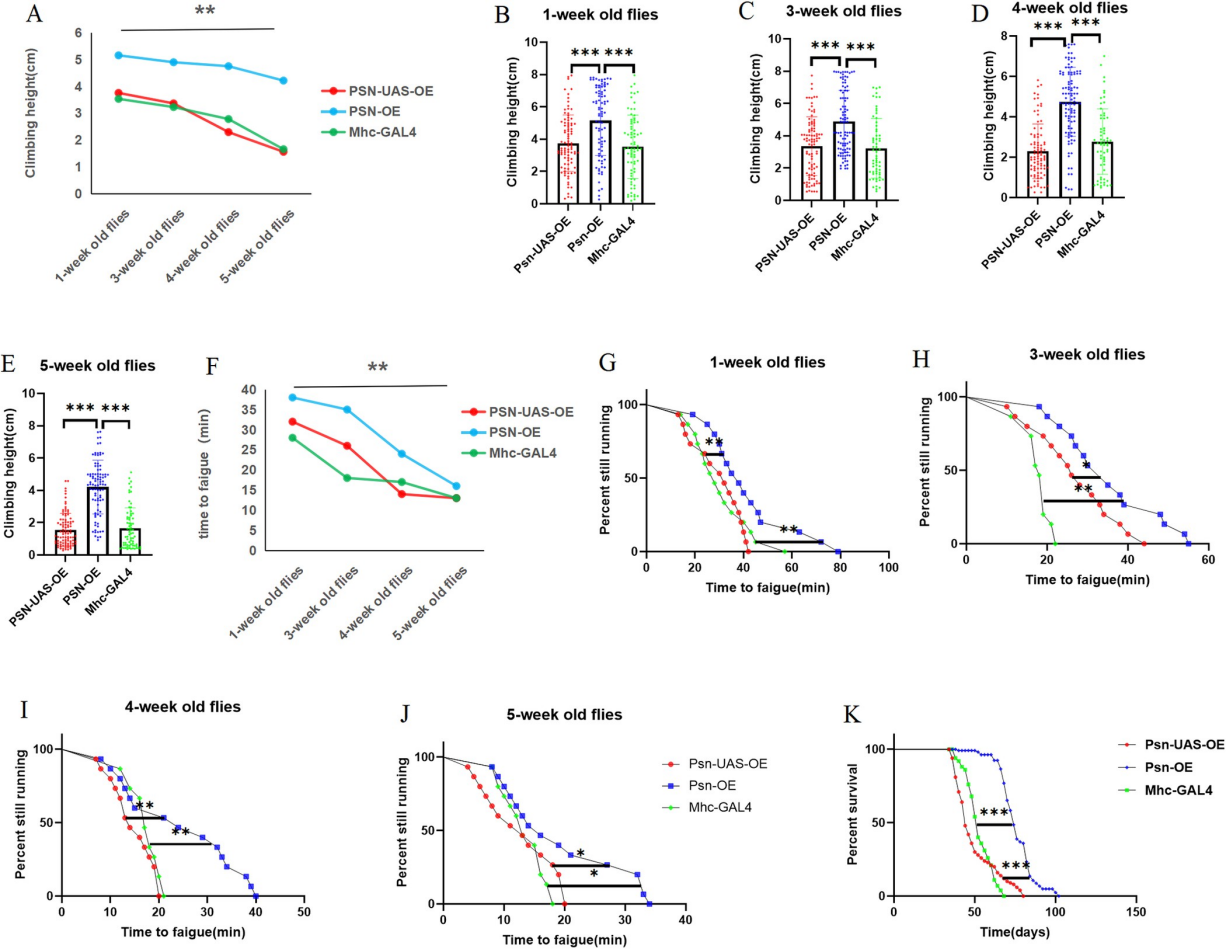
Effects of MPO on muscle function and mean lifespan in *Drosophila*. (A) Climbing height changed with age in *Drosophila*. (B) Climbing the height of 1-week-old *Drosophila* in 3 seconds. (C) Climbing the height of 3-week-old *Drosophila* in 3 seconds. (D) Climbing the height of 4-week-old *Drosophila* in 3 seconds. (E) Climbing the height of 5-week-old *Drosophila* in 3 seconds. (F) Time to fatigue changed with age in *Drosophila*. (G) Time to fatigue in 1-week-old *Drosophila*. (H) Time to fatigue in 3-week-old *Drosophila*. (I) Time to fatigue in 4-week-old *Drosophila*. (J) Time to fatigue in 5-week-old *Drosophila*. (K) The average lifespan. For the climbing index and climbing height measurement, the sample size was about 100 *Drosophila* for each group. For climbing endurance, the sample size was 15 *Drosophila* for each group. P-values for climbing endurance curves were calculated by the log-rank test. The 1-way analysis of variance (ANOVA) with least significant difference (LSD) tests was used to identify differences among the groups. Data are represented as means ± SEM. *P<0.05; **P <0.01; ***P <0.001; ns means no significant differences.

The results showed that aging caused a decrease in CH in Psn-UAS-OE *Drosophila*, PSN-OE *Drosophila*, and Mhc-GAL4 *Drosophila* (Fig 1A). At the age of 1 week, Psn-OE significantly increased the CH of *Drosophila* (t = 4.729, df = 174, P<0.01), and CH were also significantly increased compared with Mhc-GAL4 in Psn-OE *Drosophila* (t = 4.961, df = 159, P<0.01) (Fig 1B). At the age of 3 weeks, Psn-OE significantly increased the CH of *Drosophila* (t = 5.708, df = 192, P<0.01), and CH were also significantly increased compared with Mhc-GAL4 in Psn-OE *Drosophila* (t = 5.422, df = 158, P<0.01) (Fig 1C). At the age of 4 weeks, Psn-OE significantly increased the CH of *Drosophila* (t = 10.98, df = 192, P<0.01), and CH were also significantly increased compared with Mhc-GAL4 in Psn-OE *Drosophila* (t = 7.368, df = 165, P<0.01) (Fig 1D). At the age of 5 weeks, Psn-OE significantly increased the CH of *Drosophila* (t = 13.16, df = 182, P<0.01), and CH were also significantly increased compared with Mhc-GAL4 in Psn-OE *Drosophila* (t = 10.83, df = 160, P<0.01) (Fig 1E).

Moreover, the results showed that aging caused a decrease in TTF in Psn-UAS-OE *Drosophila*, PSN-OE *Drosophila*, and Mhc-GAL4 *Drosophila* (Fig 1F). At the age of 1 week, Psn-OE significantly increased the TTF of *Drosophila* (P = 0.0199), and TTF were also significantly increased compared with Mhc-GAL4 in Psn-OE *Drosophila* (P = 0.0381) (Fig 1G). At the age of 3 weeks, Psn-OE significantly increased the TTF of *Drosophila*(P = 0.0437), and TTF were also significantly increased compared with Mhc-GAL4 in Psn-OE *Drosophila* (P<0.0001) (Fig 1H). At the age of 4 weeks, Psn-OE significantly increased the TTF of *Drosophila* (P = 0.0036), and TTF were also significantly increased compared with Mhc-GAL4 in Psn-OE *Drosophila* (P = 0.0170) (Fig 1I). At the age of 5 weeks, Psn-OE significantly increased the TTF of *Drosophila* (P = 0.0325), and TTF were also significantly increased compared with Mhc-GAL4 in Psn-OE *Drosophila* (P = 0.0307) (Fig 1J).

Furthermore, Compared with Psn-UAS-OE and Mhc-GAL4, the lifespan of Psn-OE *Drosophila* were increased (P<0.0001,P<0.0001) (Fig 1K). The maximum life span of Psn-UAS-OE *Drosophila* were 80d, and the average life span were 44d; the maximum life span of Psn-OE *Drosophila* were 102d, the average life span were 74d, and the average life expectancy were 68.18%; the maximum life span of Mhc-GAL4 *Drosophila* were 68d, and the average life span were 52d, compared with that of Psn-OE *Drosophila*, the average life expectancy were -29.73%.

These results suggested that MPO improved exercise capacity and lifespan in aged *Drosophila*. However, the molecular mechanism is unknown, and to investigate the molecular mechanism examined Psn/Sirt1/PGC-1α pathway, and Psn/arm pathway.

**Fig 2:**
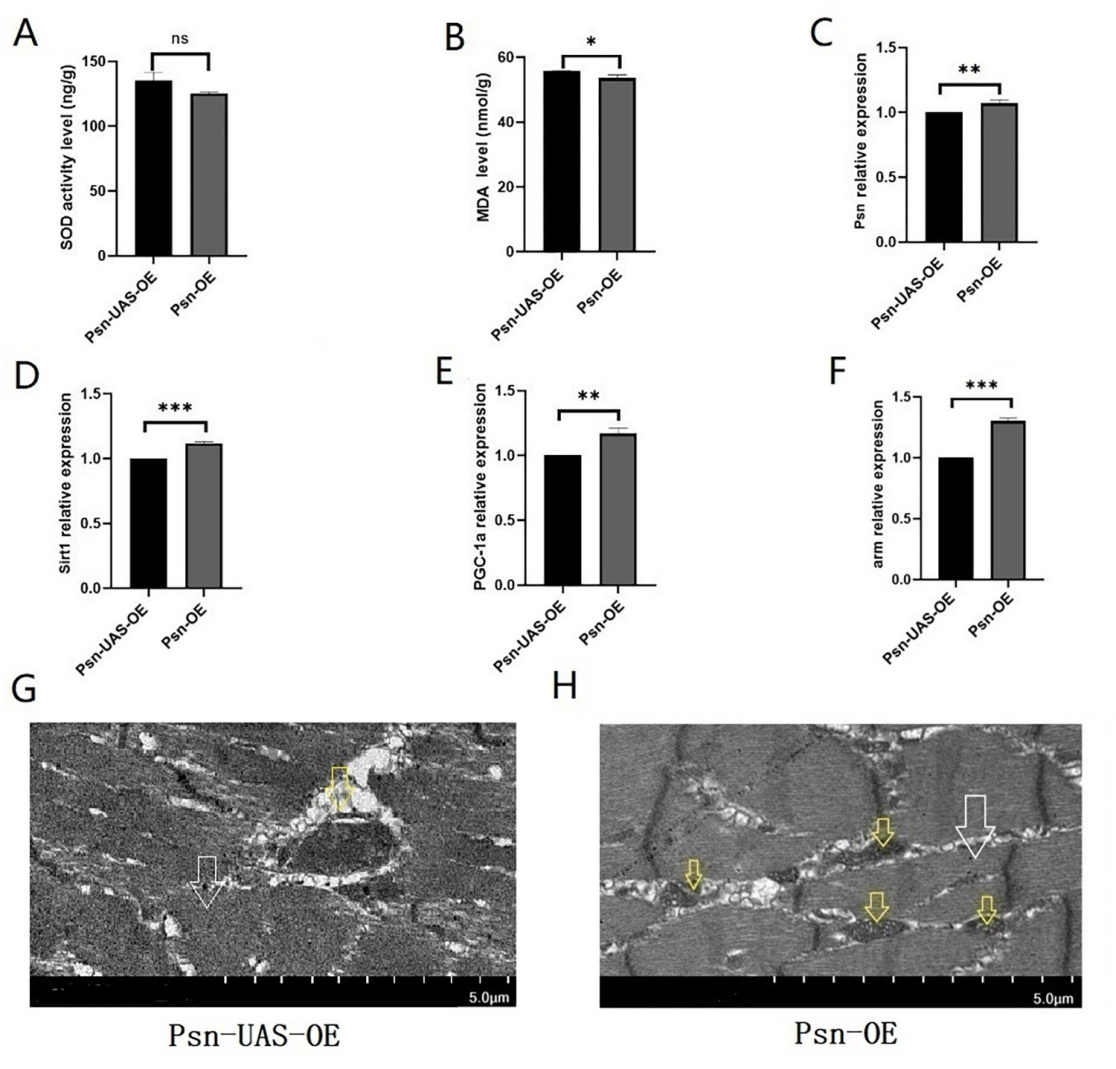
Effects of MPO on muscle function in *Drosophila*. (A) SOD activity level. (B) MDA level. (C) The expression of the Psn gene in skeletal muscle. (D) The expression of the Sirt1 gene in skeletal muscle. (E) The expression of PGC-1αgene in skeletal muscle. (F) The expression of the arm gene in skeletal muscle. (G) Transmission electron microscopy of Psn-UAS-OE *Drosophila* muscle. (H) Transmission electron microscopy of Psn-OE *Drosophila* muscle. For RT-PCR measurement and ELISA measurement, the sample size was about 50/30 *Drosophila*’ skeletal muscle for each group. The 1-way analysis of variance (ANOVA) with least significant difference (LSD) tests was used to identify differences among the groups. Data are represented as means ± SEM. *P<0.05; **P <0.01; ***P <0.001; ns means no significant differences. Scale: white line represents 5 μm. Transmission electron microscopy images show that MPO increases the number of mitochondria and myogenic fibers. Yellow arrows indicate mitochondria and white arrows indictea myogenic fibers.

The results showed that in 5 weeks *Drosophila*, compared with Psn-UAS-OE, the relative expressions of Psn in the muscle of Psn-OE Drosophila were increased (t = 5.500, df = 4, P<0.01) (Fig 2C), and it also increased the relative expression of the Sirt1 gene, the PGC-1α gene, and the arm gene (t = 13.23, df = 4, P<0.01, t = 7.361, df = 4, P<0.01,t = 20.88, df = 4, P<0.01) (Fig 2D–2F). However, Psn-UAS-OE did not significantly change the SOD level (t = 1.896, df = 4, P>0.05) (Fig 2A) Finally, the results showed MPO significantly decreased the MDA level (t = 4.062, df = 4, P<0.05) (Fig 2B). In addition, transmission electron microscopy images show that MPO can increase the number of mitochondrial and promote the integrity of myogenic fibers and Z-wires (Fig 2G and 2H).

These results suggested that MPO improves age-related decline in skeletal muscle and lifespan in *Drosophila* by reducing muscles’ oxidative stress and activating Psn/Sirt1/PGC-1α pathway and Psn/arm pathway.

### MPO combined with endurance exercise further improves age-related decline in skeletal muscle and lifespan in *Drosophila*

Studies have shown that endurance exercise training reduces oxidative damage to muscles and may improve climbing ability and longevity in *Drosophila* to some extent [28]. However, it remains unclear whether the molecular regulation of skeletal muscle Psn gene overexpression involved in exercise delayed skeletal muscle aging.

**Fig 3:**
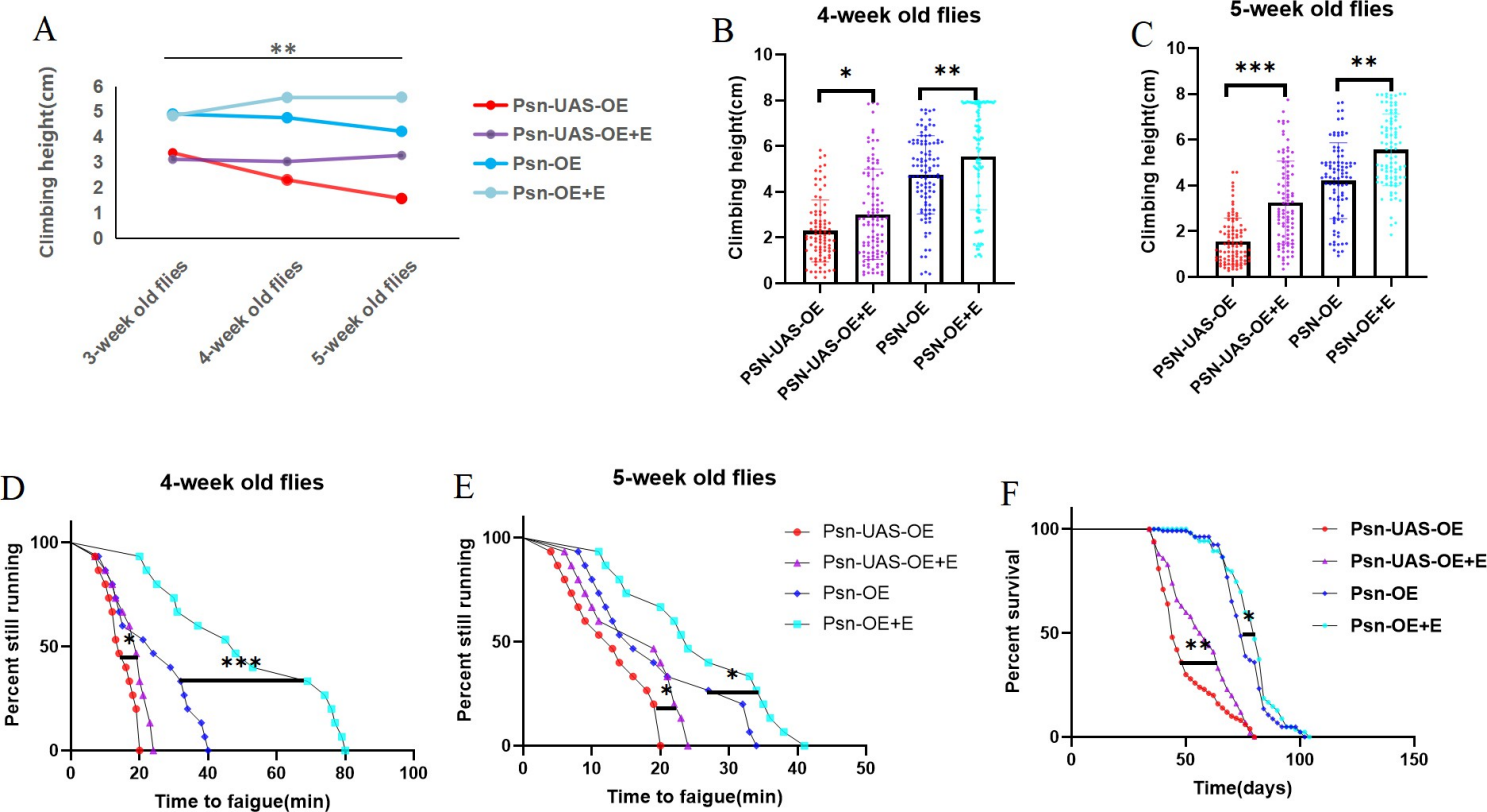
Effects of endurance exercise on muscle function and mean lifespan in *Drosophila*. Climbing height changed with age in *Drosophila*. (B) Climbing the height of 4-week-old *Drosophila* in 3 seconds. (C) Climbing the height of 5-week-old *Drosophila* in 3 seconds. (D) Time to fatigue in 4-week-old *Drosophila*. (E) Time to fatigue in 5-week-old *Drosophila*. (F) The average lifespan. For the climbing index and climbing height measurement, the sample size was about 100 *Drosophila* for each group. For climbing endurance, the sample size was 15 *Drosophila* for each group. P-values for climbing endurance curves were calculated by the log-rank test. The 1-way analysis of variance (ANOVA) with least significant difference (LSD) tests was used to identify differences among the groups. Data are represented as means ± SEM. *P<0.05; **P <0.01; ***P <0.001; ns means no significant differences.

We performed exercise intervention on Psn-UAS-OE and Psn-OE *Drosophila*, and the results showed that CH did not decrease with aging after exercise, but showed an upward trend (Fig 3A). The results showed that at the age of 4 weeks, Psn-UAS-OE+E *Drosophila* CH were significantly increased (t = 2.937, df = 191, P<0.05), and Psn-OE+E *Drosophila* CH were also significantly increased (t = 2.810, df = 203, P<0.01) (Fig 3B). At the age of 5 weeks, Psn-UAS-OE+E *Drosophila* CH were significantly increased (t = 7.924, df = 185, P<0.01), and Psn-OE+E *Drosophila* CH were also significantly increased (t = 5.665, df = 180, P<0.01) (Fig 3C).

In addition, at the age of 4 weeks, Psn-UAS-OE+E *Drosophila* TTF were significantly increased (P = 0.0263), and Psn-OE+E *Drosophila* TTF were also significantly increased (P = 0.0004) (Fig 3D). At the age of 5 weeks, Psn-UAS-OE+E *Drosophila* TTF were significantly increased (P = 0.0235), and Psn-OE+E *Drosophila* TTF were also significantly increased (P = 0.0377) (Fig 3E).

Moreover, Compared with Psn-UAS-OE, the lifespan of Psn-UAS-OE+E Drosophila were increased (P = 0.0049), and compared with Psn-OE, the lifespan of Psn-OE+E Drosophila were increased (P = 0.0375) (Fig 3F). The maximum life span of Psn-UAS-OE *Drosophila* were 80d and the average life span were 44d; the maximum life span of Psn-UAS-OE+E *Drosophila* were 78d, the average life span was 57d, and the average life expectancy were 29.55%. The maximum life span of Psn-OE *Drosophila* were 102d, the average life span were 74d; the maximum life span of Psn-UAS-OE+E *Drosophila* were 104d, the average life span were 80d, and the average life expectancy were 8%.

These results indicated that both endurance exercise and MPO combined with endurance exercise could improve exercise capacity and lifespan in aged *Drosophila*. However, the molecular mechanism is unknown, and to investigate the molecular mechanism examined the Psn/Sirt1/PGC-1α pathway and Psn/arm pathway.

**Fig 4:**
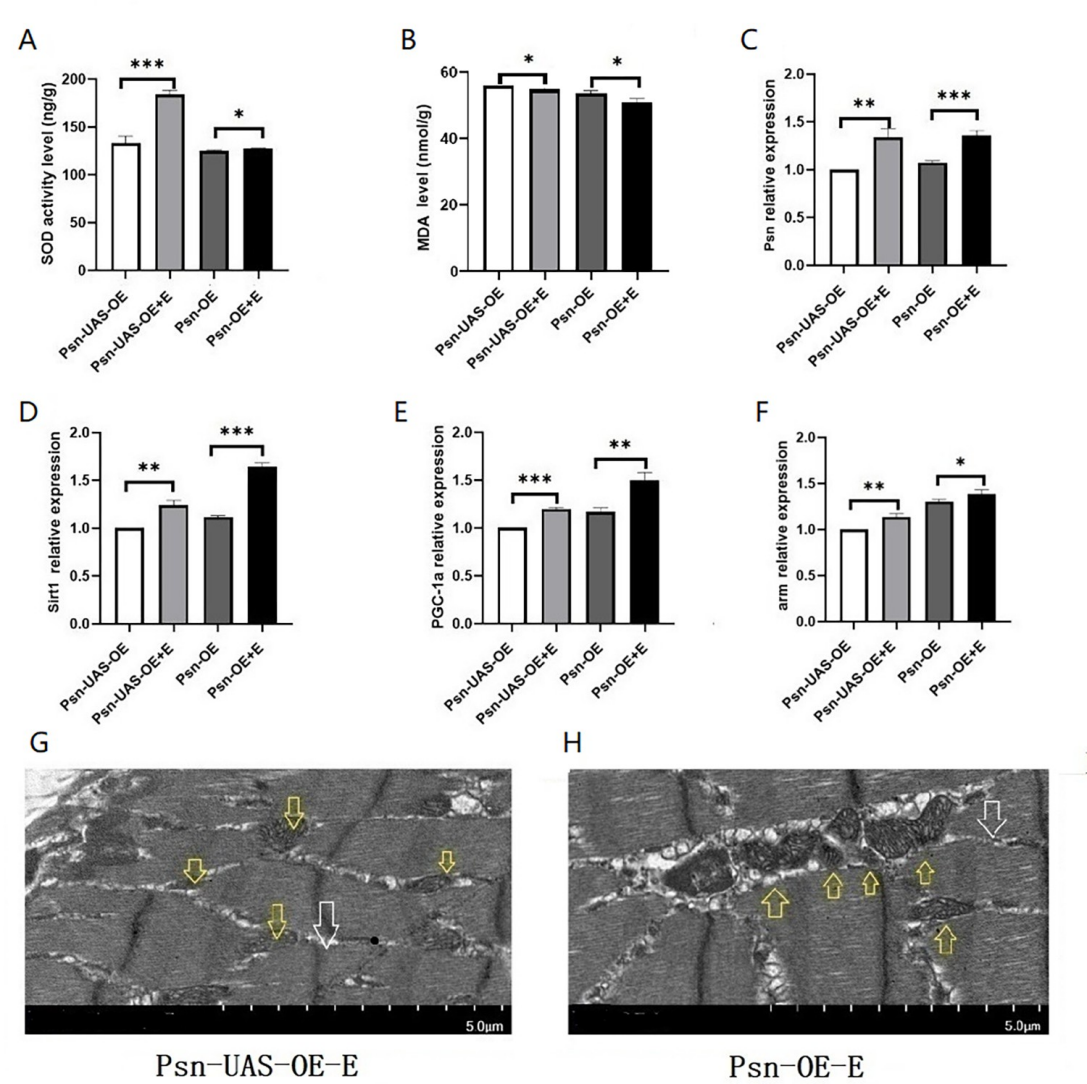
Effects of endurance exercise on muscle function in *Drosophila*. SOD activity level. (B) MDA level. (C) The expression of the Psn gene in skeletal muscle. (D) The expression of the Sirt1 gene in skeletal muscle. (E) The expression of PGC-1αgene in skeletal muscle. (F) The expression of the arm gene in skeletal muscle. (G) Transmission electron microscopy of Psn-UAS-OE-E *Drosophila* muscle. (H) Transmission electron microscopy of Psn-OE-E *Drosophila* muscle. For the climbing index and climbing height measurement, the sample size was about 100 *Drosophila* for each group. For RT-PCR measurement and ELISA measurement, the sample size was about 50/30 *Drosophila*’ skeletal muscle for each group. The 1-way analysis of variance (ANOVA) with least significant difference (LSD) tests was used to identify differences among the groups. Data are represented as means ± SEM. *P<0.05; **P <0.01; ***P <0.001; ns means no significant differences. Scale: white line represents 5 μm. Transmission electron microscopy images show that endurance exercise increases the number of mitochondria and myogenic fibers. Yellow arrows indicate mitochondria and white arrows indicate myogenic fibers.

Our results showed that in 5 weeks *Drosophila*, compared with Psn-UAS-OE, the relative expressions of Psn in the muscle of Psn-UAS-OE+E Drosophila were increased (t = 6.543, df = 4, P<0.01),and compared with Psn-OE, the relative expressions of Psn in the muscle of Psn-OE+E Drosophila also were increased (t = 9.015, df = 4, P<0.01) (Fig 4C). In addition, endurance exercise significantly increased the relative expression of the Sirt1 gene, the PGC-1α gene, and the arm gene in muscle of Psn gene *Drosophila* (t = 8.314, df = 4, P<0.01, t = 20.00, df = 4, P<0.01, t = 6.740, df = 4, P<0.01), and it also significantly increased the relative expression of the Sirt1 gene, the PGC-1α gene, and the arm gene in muscle of MPO *Drosophila* (t = 23.97, df = 4, P<0.01, t = 6.390, df = 4, P<0.01, t = 2.795, df = 4, P<0.05) (Fig 3D–3F).

Moreover, Psn-UAS-OE+E *Drosophila* significantly increased the SOD level (t = 10.51, df = 4, P<0.01), and Psn-OE+E *Drosophila* also significantly increased the SOD level (t = 3.134, df = 4, P<0.05) (Fig 4A). Finally, the results showed that Psn-UAS-OE+E *Drosophila* significantly decreased the MDA level (t = 3.224, df = 4, P<0.05), and Psn-OE+E *Drosophila* also significantly decreased the MDA level (t = 3.359, df = 4, P<0.05) (Fig 4B). In addition, transmission electron microscopy images show that endurance exercise can increase the number of mitochondrial and promote the integrity of myogenic fibers and Z-wires (Fig 3G and 3H).

These results suggested that MPO and MPO combined with endurance exercise improve age-related decline in skeletal muscle and lifespan in *Drosophila* by activating Psn/Sirt1/PGC-1α pathway and Psn/arm pathway and reducing oxidative stress.

### MPK improves age-related decline in skeletal muscle and lifespan in *Drosophila*

**Fig 5:**
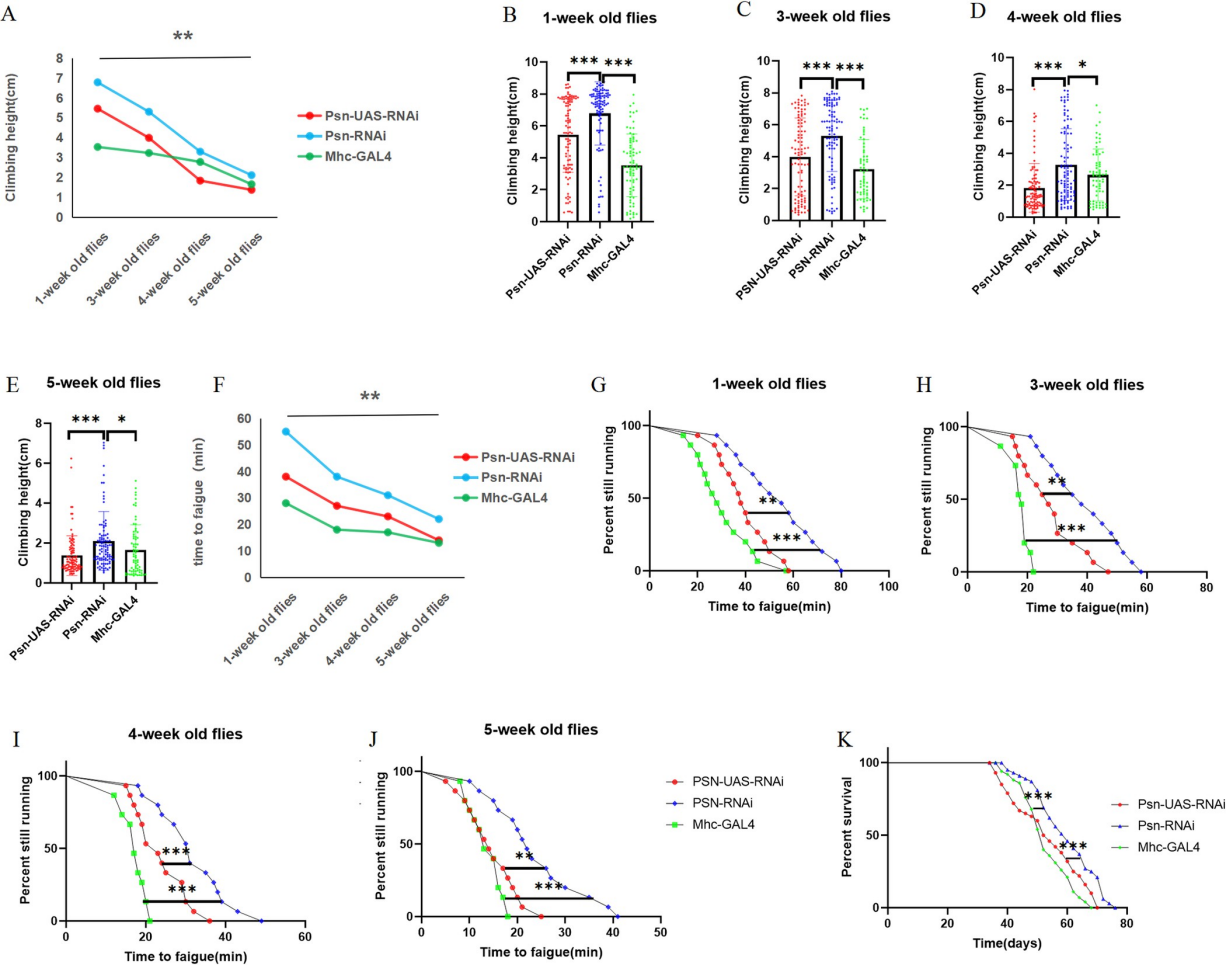
Effects of MPK on muscle function and mean lifespan in *Drosophila*. (A) Climbing height changed with age in *Drosophila*. (B) Climbing the height of 1-week-old *Drosophila* in 3 seconds. (C) Climbing the height of 3-week-old *Drosophila* in 3 seconds. (D) Climbing the height of 4-week-old *Drosophila* in 3 seconds. (E) Climbing the height of 5-week-old *Drosophila* in 3 seconds. (F) Time to fatigue changed with age in *Drosophila*. (G) Time to fatigue in 1-week-old *Drosophila*. (H) Time to fatigue in 3-week-old *Drosophila*. (I) Time to fatigue in 4-week-old *Drosophila*. (J) Time to fatigue in 5-week-old *Drosophila*. (K) The average lifespan. For the climbing height and lifespan measurement, the sample size was about 100 *Drosophila* for each group. For climbing endurance, the sample size was 15 *Drosophila* for each group. P-values for climbing endurance curves were calculated by the log-rank test. The 1-way analysis of variance (ANOVA) with least significant difference (LSD) tests was used to identify differences among the groups. Data are represented as means ± SEM. *P<0.05; **P <0.01; ***P <0.001; ns means no significant differences.

In this study, MPK was constructed by Psn-UAS/Mhc-GAL4 system. The results showed that aging caused a decrease in CH inPsn-UAS-RNAi *Drosophila*, PSN-RNAi *Drosophila*, and Mhc-GAL4 *Drosophila* (Fig 5A). At the age of 1 week, Psn-RNAi significantly increased the CHof *Drosophila* (t = 4.299, df = 198, P<0.01), and CH were also significantly increased compared with Mhc-GAL4 in Psn-RNAi *Drosophila* (t = 10.89, df = 175, P<0.01) (Fig 5B). At the age of 3 weeks, Psn-RNAi significantly increased the CH of *Drosophila* (t = 3.940, df = 194, P<0.01), and CH were also significantly increased compared with Mhc-GAL4 in Psn-RNAi *Drosophila* (t = 6.127, df = 158, P<0.01) (Fig 5C). At the age of 4 weeks, Psn-RNAi significantly increased the CH of *Drosophila* (t = 5.357, df = 198, P<0.01), and CH were also significantly increased compared with Mhc-GAL4 in Psn-RNAi *Drosophila* (t = 2.027, df = 166, P<0.05) (Fig 5D). At the age of 5 weeks, Psn-RNAi significantly increased the CH of *Drosophila* (t = 4.138, df = 198, P<0.01), and CH were also significantly increased compared with Mhc-GAL4 in Psn-RNAi *Drosophila* (t = 2.125, df = 170, P<0.05) (Fig 5E).

Moreover, the results showed that aging caused a decrease in TTF in Psn-UAS-RNAi *Drosophila*, Psn-RNAi *Drosophila*, and Mhc-GAL4 *Drosophila* (Fig 5F). At the age of 1 week, Psn-RNAi significantly increased the TTF of *Drosophila* (P = 0.0041), and TTF were also significantly increased compared with Mhc-GAL4 in Psn-RNAi *Drosophila* (P<0.0001) (Fig 5G). At the age of 3 weeks, Psn-RNAi significantly increased the TTF (P = 0.0071), and TTF were also significantly increased compared with Mhc-GAL4 in Psn-RNAi *Drosophila* (P<0.0001) (Fig 5H). At the age of 4 weeks, Psn-RNAi significantly increased the TTF (P = 0.0051), and TTF were also significantly increased compared with Mhc-GAL4 in Psn-RNAi *Drosophila* (P<0.0001) (Fig 5I). At the age of 5 weeks, Psn-RNAi significantly increased the TTF (P = 0.0011), and TTF were also significantly increased compared with Mhc-GAL4 in Psn-RNAi *Drosophila* (P<0.0001) (Fig 5J).

Furthermore, Compared with Psn-UAS-RNAi and Mhc-GAL4, the lifespan of Psn-RNAi Drosophila were increased (P<0.0001, P<0.0001) (Fig 5K). The maximum lifespan of Psn-UAS-RNAi *Drosophila* were 70d, and the average lifespan were 53d; the maximum lifespan of Psn-RNAi *Drosophila* were 76d, the average life span were 60d, and the average life expectancy were 13.2%; the maximum life span of Mhc-GAL4 *Drosophila* were 68d, and the average life span were 52d, compared with that of Psn-RNAi *Drosophila*, the average life expectancy were -13.33%.

**Fig 6:**
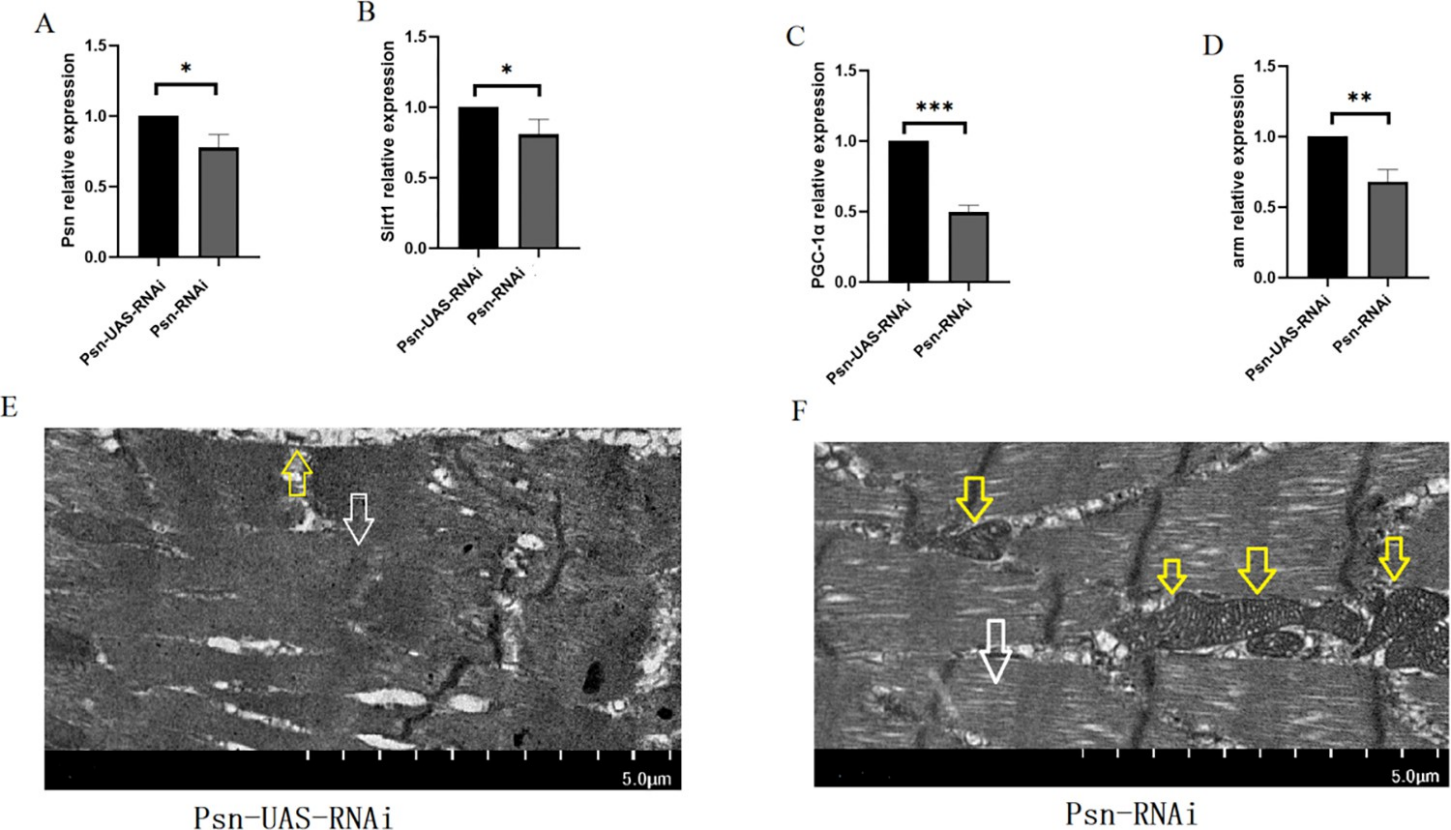
Effects of MPK on muscle function in *Drosophila*. (A) The expression of the Psn gene in skeletal muscle. (B) The expression of the Sirt1 gene in skeletal muscle. (C) The expression of PGC-1αgene in skeletal muscle. (D) The expression of the arm gene in skeletal muscle. (E) Transmission electron microscopy of Psn-UAS-RNAi *Drosophila* muscle. (F) Transmission electron microscopy of Psn-RNAi *Drosophila* muscle. For RT-PCR measurement, the sample size was about 50 *Drosophila*’ skeletal muscle for each group. The 1-way analysis of variance (ANOVA) with least significant difference (LSD) tests was used to identify differences among the groups. Data are represented as means ± SEM. *P<0.05; **P <0.01; ***P <0.001; ns means no significant differences. Scale: white line represents 5 μm. Transmission electron microscopy images show that MPO increases the number of mitochondria and myogenic fibers. Yellow arrows indicate mitochondria and white arrows indicate myogenic fibers.

The results showed that in 5-week-old *Drosophila*, compared with Psn-UAS-RNAi, the relative expressions of Psn in the muscle of Psn-RNAi Drosophila were decreased by 0.78 times (t = 4.163, df = 4, P<0.05) (Fig 6A), and it also decreased the relative expression of the Sirt1 gene, the PGC-1α gene, and the arm gene (t = 3.110, df = 4, P<0.05, t = 17.67, df = 4, P<0.01, t = 6.358, df = 4, P<0.01) (Fig 6B–6D). In addition, transmission electron microscopy images show that MPK can increase the number of mitochondrial and promote the integrity of myogenic fibers and Z-wires (Fig 6E and 6F).

The results showed that when the Psn gene is knockdown by 0.78 times, it could reduce the adaptability of Sirt expression, PGC-1α expression, and arm expression and improve the exercise ability and lifespan in aged *Drosophila*.

### MPK combined with endurance exercise further improves age-related decline in skeletal muscle in *Drosophila*

**Fig 7:**
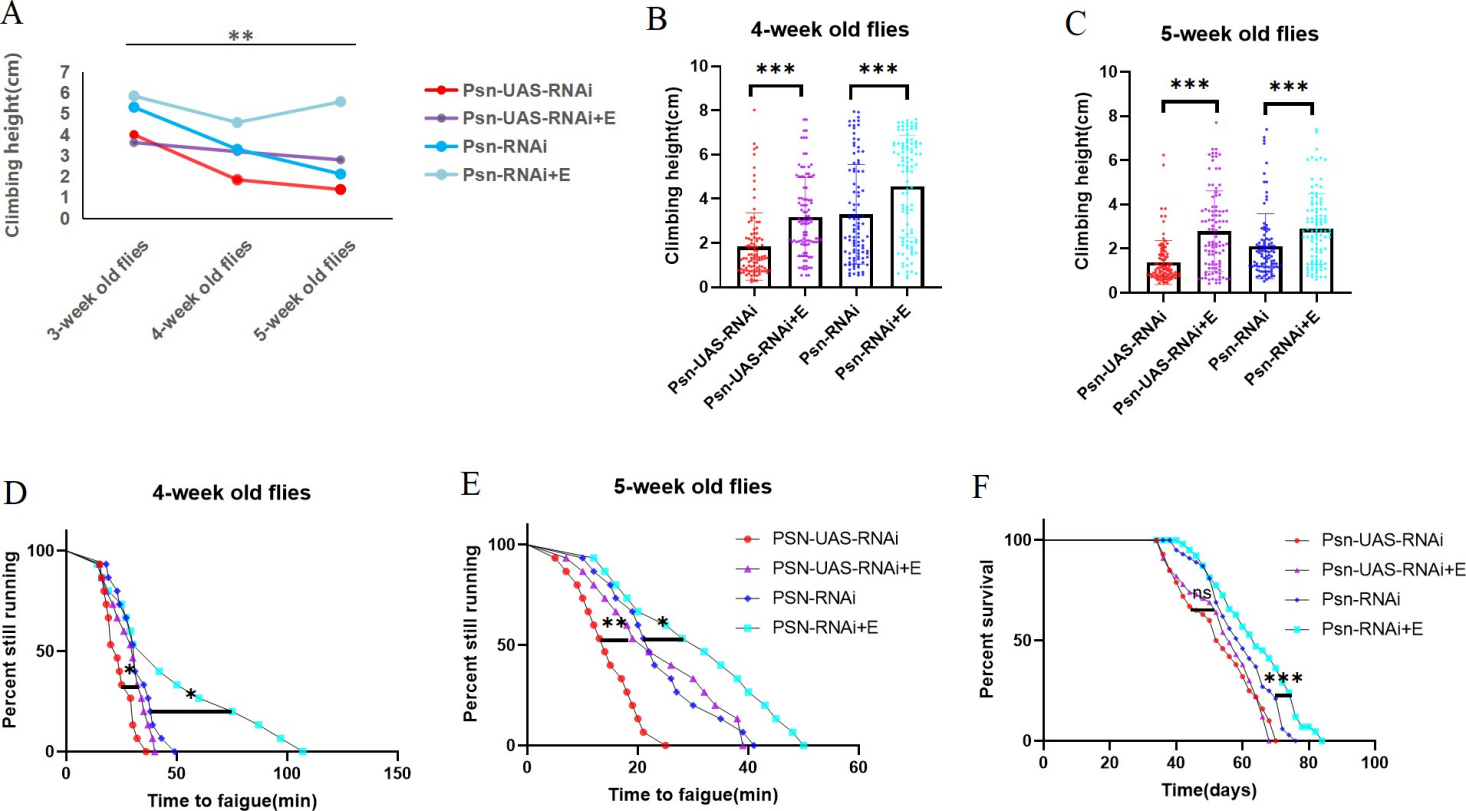
Effects of endurance exercise on muscle function and mean lifespan in *Drosophila*. (A) Climbing height changed with age in *Drosophila*. (B) Climbing the height of 4-week-old *Drosophila* in 3 seconds. (C) Climbing the height of 5-week-old *Drosophila* in 3 seconds. (D) Time to fatigue in 4-week-old *Drosophila*. (E) Time to fatigue in 5-week-old *Drosophila*. (F) The average lifespan. For the climbing height and lifespan measurement, the sample size was about 100 *Drosophila* for each group. For climbing endurance, the sample size was 15 *Drosophila* for each group. P-values for climbing endurance curves were calculated by the log-rank test. The 1-way analysis of variance (ANOVA) with least significant difference (LSD) tests was used to identify differences among the groups. Data are represented as means ± SEM. *P<0.05; **P <0.01; ***P <0.001; ns means no significant differences.

In this study, it remains unclear whether the molecular regulation of skeletal muscle Psn gene knockdown involved in exercise delayed skeletal muscle aging. We performed exercise intervention on Psn-UAS-RNAi and Psn-RNAi *Drosophila*, and the results showed that CH did not decrease with aging after exercise, but showed an upward trend (Fig 7A). at the age of 4 weeks, Psn-UAS-RNAi+E *Drosophila* CH were significantly increased (t = 5.716, df = 198, P<0.01), and Psn-RNAi+E *Drosophila* CH were also significantly increased (t = 3.957, df = 198, P<0.01) (Fig 7B). at the age of 5 weeks, Psn-UAS-RNAi+E *Drosophila* CH were significantly increaseds (t = 6.803, df = 198, P<0.01), and Psn-RNAi+E *Drosophila* CH were also significantly increased (t = 3.598, df = 198, P<0.01) (Fig 7C).

In addition, at the age of 4 weekss, Psn-UAS-RNAi+E *Drosophila* TTF were significantly increased (P = 0.0439), and Psn-RNAi+E *Drosophila* TTF were also significantly increased (P = 0.0454) (Fig 7D). At the age of 5 weekss, Psn-UAS-RNAi+E *Drosophila* TTF were significantly increased (P = 0.0036), and Psn-RNAi+E *Drosophila* TTF were also significantly increased (P = 0.0474) (Fig 7E).

However, compared with Psn-UAS-RNAi, the lifespan of Psn in the muscle of Psn-UAS-RNAi+E Drosophila were not significantly increased (P = 0.7338), but compared with Psn-RNAi, the lifespan of Psn-RNAi+E Drosophila were increased (P<0.0001) (Fig 7F). The maximum life span of Psn-UAS-RNAi+E *Drosophila* were 68d, the average life span were 56d. The maximum life span of Psn-RNAi+E *Drosophila* were 84d, the average life span were 64d, and the average life expectancy were 6.7%.

**Fig 8:**
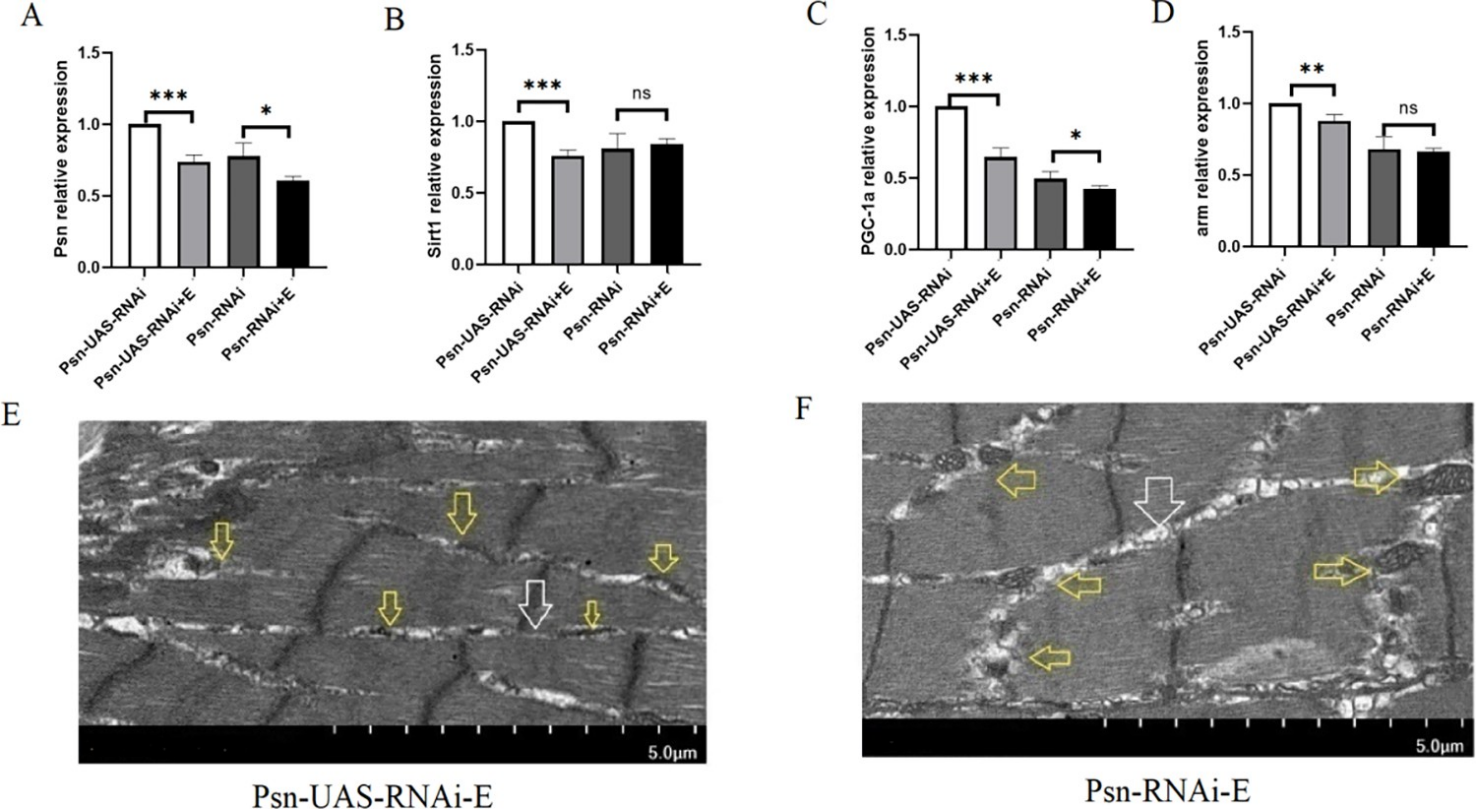
Effects of endurance exercise on muscle function in *Drosophila*. (A)The expression of the Psn gene in skeletal muscle. (B) The expression of the Sirt1 gene in skeletal muscle. (C) The expression of PGC-1αgene in skeletal muscle. (D) The expression of the arm gene in skeletal muscle. (E) Transmission electron microscopy of Psn-UAS-RNAi+E *Drosophila* muscle. (F) Transmission electron microscopy of Psn-RNAi+E *Drosophila* muscle. For the climbing height and lifespan measurement, the sample size was about 100 *Drosophila* for each group. For RT-PCR measurement, the sample size was about 50 *Drosophila*’ skeletal muscle for each group. The 1-way analysis of variance (ANOVA) with least significant difference (LSD) tests was used to identify differences among the groups. Data are represented as means ± SEM. *P<0.05; **P <0.01; ***P <0.001; ns means no significant differences. Scale: white line represents 5 μm. Transmission electron microscopy images show that endurance exercise increases the number of mitochondria and myogenic fibers. Yellow arrows indicate mitochondria and white arrows indicate myogenic fibers.

Our results showed that in 5 weeks *Drosophila*, compared with Psn-UAS-RNAi, the relative expressions of Psn in the muscle of Psn-UAS-RNAi+E Drosophila were decreased (t = 8.888, df = 4, P<0.01), and compared with Psn-RNAi, the relative expressions of Psn in the muscle of Psn-RNAi+E Drosophila also were decreased (t = 3.010, df = 4, P<0.05) (Fig 8A). In addition, endurance exercise significantly decreased the relative expression of the Sirt1 gene, the PGC-1α gene, and the arm gene in Psn gene *Drosophila* (t = 10.39, df = 4, P<0.01, t = 9.636, df = 4, P<0.01, t = 4.768, df = 4, P<0.01), and it also significantly decreased the relative expression of the PGC-1α gene in MPK *Drosophila* (t = 2.264, df = 4, P<0.05) (Fig 8B–8D). In addition, transmission electron microscopy images show that endurance exercise can increase the number of mitochondrial and promote the integrity of myogenic fibers and Z-wires (Fig 8E and 8F).

These results suggested that MPK combined with endurance exercise further improved exercise capacity and further delayed age-related decline in skeletal muscle and lifespan in *Drosophila*.

## Discussion

There is a very close relationship between Psn and aging. Previous studies have shown that during aging, the presence of Psn is necessary for cortical excitatory neurons to survive [29]. Moreover, Psn also plays a very critical protective role in the survival of cortical interneurons during the aging process [30]. Psn mutations also induce the development of age-dependent neurodegenerative diseases such as Alzheimer’s disease and dilated cardiomyopathy [31]. PS1 and PS2 are highly expressed in neurons throughout development [32]. It has been shown that PS1 delays cellular senescence and reduces the onset of oxidative stress, which is achieved by regulating the cell cycle through the modulation of the Wnt/arm pathway [33]. For the Wnt/arm pathway, activation of this pathway not only slows down aging and prevents age-dependent neurodegenerative diseases, but also regulates the growth and developmental processes of the organism [34]. Studies have shown that Sirt1 can regulate cellular senescence and organismal aging as a way to exert anti-aging effects [35]. Sirt1 expression decreases with age, and increasing Sirt1 expression extends lifespan [36]. The activity of Sirt1 is inhibited by the reaction product nicotinamide, and cellular metabolism and redox status also influence Sirt1 activity [37]. Therefore, one way to extend lifespan is to activate Sirt1 to upregulate PGC-1α activity by increasing intracellular NAD concentration, which ultimately promotes mitochondrial biogenesis, oxidative metabolism, and types I myofibres, thereby delaying skeletal muscle aging and lifespan [38]. In addition, cell cycle and apoptosis can be regulated by activating the arm pathway, thereby slowing down aging [39].

We discover that the enhanced exercise ability and increased lifespan of the Psn gene *Drosophila* are associated with the activation of the Psn/Sirt1/PGC-1α pathway and Psn/arm pathway. The findings suggest that gene overexpression or knockdown has become a classical approach to confirming gene function [40]. To confirm this hypothesis, we constructed MPO and MPK *Drosophila* using the Psn-UAS/Mhc-GAL4 system. In experiments, there is growing evidence that MPO significantly increased exercise capacity, lifespan, heart period, muscle SOD activity, Psn gene, Sirt1 gene, PGC-1α gene, and arm gene relative expression in aged *Drosophila*, and they significantly decreased muscle MDA levels. Thus, current evidence confirmed that MPO delays skeletal muscle aging and lifespan by reducing muscles’ oxidative stress and activating the muscle Psn/Sirt1/PGC-1α pathway and Psn/arm pathway.

As we age, the body experiences irreversible aging due to a gradual decline in the body’s antioxidant defenses [41]. Therefore, the body’s antioxidant defenses are a key way to slow down aging [42]. Existing research has proven that performing endurance exercise training can be very beneficial to the organism by exerting effective antioxidant and anti-aging key effects [14]. Existing studies have shown that regular endurance training is one of the simple, effective, and easy ways to improve athletic performance to combat oxidation of the body, and mitigate aging [43]. Endurance exercise training also enhances the body’s ability to resist oxidative stress by increasing muscle SOD activity and decreasing muscle MDA levels [44]. In addition, some findings suggest that endurance exercise training also leads to an increase in mitochondrial density in muscles and neurons, because endurance exercise training activates PGC-1α activity by upregulating Sirt1 levels [45]. It has been found that mitochondria play a very important role in the beneficial effects of endurance exercise training on the body [46]. Studies have shown that mitochondria and exercise also interact, with muscle mitochondria regulating skeletal muscle mass and function, which, in turn, they regulate through exercise [47]. Endurance exercise training not only improves mitochondrial biogenesis and respiration but also induces mitochondrial plasticity [48]. Exercise also enhances antioxidant capacity and mitochondrial affinity for oxygen, delaying aging [49]. Upregulation of motor-regulated PGC-1α levels also mediates mitochondrial biogenesis [50]. It has also been shown that PGC-1α is also an exercise-responsive gene and that its increased gene expression increases the ability of *Drosophila* to climb and improves cardiac function [51]. The arm, on the other hand, is necessary for the proper functioning of muscles during endurance exercise [52]. To further confirm that the skeletal muscle Psn gene is involved in the molecular regulation of exercise in delaying skeletal muscle aging, we combined endurance exercise with MPO. In this experiment, we found exercise significantly increased exercise capacity, lifespan, muscle SOD activity, Psn expression, Sirt1 expression, PGC-1α expression, and arm expression in aged *Drosophila*, and they significantly decreased muscle MDA levels. In addition, MPO combined with exercise further improved exercise capacity and lifespan. Thus, current evidence confirmed that the exercise delayed skeletal muscle aging and lifespan by reducing muscles’ oxidative stress and activating muscle Psn/Sirt1/PGC-1α pathway and Psn/arm pathway, and MPO combined with exercise further delayed skeletal muscle aging and lifespan by further reducing muscles’ oxidative stress and activating muscle Psn/Sirt1/PGC-1α pathway and Psn/arm pathway.

The study showed that Knockdown of the PS1 gene prevents normal cell growth and leads to a decrease in cell activity [53]. Knockdown of Psn in adult neurons leads to the negative effects of reduced motility, shorter lifespan, increased apoptosis, and age-dependent neurodegenerative lesion development [54]. PS2 knockdown will also play an important role in the treatment of gliomas [55]. However, our experiment found that when the Psn gene is knockdown by 0.78 times, it significantly increases exercise capacity and lifespan and significantly decreases Psn expression, Sirt1 expression, PGC-1α expression, and arm expression in aged *Drosophila*. We also found that endurance exercise increased exercise ability and significantly decreased the Psn gene, Sirt1 gene, PGC-1αgene, and arm gene relative expression in aged *Drosophila*. In addition, MPK combined with endurance exercise further improves exercise ability and lifespan. Thus, current evidence confirmed that when the Psn gene is knockdown by 0.78 times, it can delay skeletal muscle aging and lifespan, and MPK can decrease the adaptability of Psn expression, Sirt expression, PGC-1α expression, and arm expression in aged *Drosophila*. And MPK combined with exercise further delayed skeletal muscle aging and lifespan.

## Conclusion

Current evidence confirmed that MPO and exercise delayed skeletal muscle aging and lifespan by reducing muscles’ oxidative stress and activating muscle Psn/Sirt1/PGC-1α pathway and Psn/arm pathway, and MPO combined with exercise further delayed skeletal muscle aging and lifespan by further reducing muscles’ oxidative stress and activating muscle Psn/Sirt1/PGC-1α pathway and Psn/arm pathway. Moreover, appropriate MPK(0.78 times) also delayed skeletal muscle aging and longevity, and MPK combined with exercise further delayed skeletal muscle aging and longevity.

## Supporting information

S1 Data (XLSX)

## Abbreviations

Psn: Presenilin
MPO: muscle Psn overexpression
MPk: muscle Psn knockdown
SOD: superoxide dismutase
Sirt1: sirtuin 1
PGC-1α: Peroxisome proliferator-activated receptor-gamma coactivator -1alpha
arm, armadillo
MDA: malondialdehyde
NAD: Nicotinamide Adenine Dinucleotide
qRT-PCR: quantificational rt-PCR
ELISA: enzyme linked immunosorbent assay
OE: overexpression
E: exercise
CH: climbing height
TTF: time to fatigue
